# West Nile Virus Capsid Protein Interacts With Biologically Relevant Host Lipid Systems

**DOI:** 10.3389/fcimb.2019.00008

**Published:** 2019-02-06

**Authors:** Ana S. Martins, Filomena A. Carvalho, André F. Faustino, Ivo C. Martins, Nuno C. Santos

**Affiliations:** Instituto de Medicina Molecular, Faculdade de Medicina, Universidade de Lisboa, Lisbon, Portugal

**Keywords:** West Nile virus, lipid droplets, lipoproteins, atomic force microscopy, force spectroscopy, dynamic light scattering, zeta potential

## Abstract

West Nile and dengue viruses are closely related flaviviruses, originating mosquito-borne viral infections for which there are no effective and specific treatments. Their capsid proteins sequence and structure are particularly similar, forming highly superimposable α-helical homodimers. Measuring protein-ligand interactions at the single-molecule level yields detailed information of biological and biomedical relevance. In this work, such an approach was successfully applied on the characterization of the West Nile virus capsid protein interaction with host lipid systems, namely intracellular lipid droplets (an essential step for dengue virus replication) and blood plasma lipoproteins. Dynamic light scattering measurements show that West Nile virus capsid protein binds very low-density lipoproteins, but not low-density lipoproteins, and this interaction is dependent of potassium ions. Zeta potential experiments show that the interaction with lipid droplets is also dependent of potassium ions as well as surface proteins. The forces involved on the binding of the capsid protein with lipid droplets and lipoproteins were determined using atomic force microscopy-based force spectroscopy, proving that these interactions are K^+^-dependent rather than a general dependence of ionic strength. The capsid protein interaction with host lipid systems may be targeted in future therapeutic strategies against different flaviviruses. The biophysical and nanotechnology approaches employed in this study may be applied to characterize the interactions of other important proteins from different viruses, in order to understand their life cycles, as well as to find new strategies to inhibit them.

## Introduction

West Nile virus (WNV) is a *Flavivirus* closely related to Dengue (DENV) and Zika (ZIKV) viruses. It was first isolated in Uganda in 1937 (Kilpatrick, 2011), and since then became endemic across Tropical Africa, Southern Asia and Northern Australia, with episodic occurrences in Europe (Kilpatrick, 2011). Despite its severity, WNV infection raised little concern until an extremely virulent strain appeared in North America, at the turn of the millennium (Reiter, 2010; Rossi et al., 2010; Kilpatrick, 2011). The virus is transmitted to humans by the bite of *Culex* spp. mosquito vectors feeding on infected birds, with migratory birds constituting the major transmission vehicle (Reisen, 2010; Reiter, 2010; Kilpatrick, 2011). In 2012, there was a resurgence in North America (5,674 and 428 clinical human cases reported in the USA and Canada, respectively) and in Europe and neighbor countries (937 cases) (Gray and Webb, 2014). In 2013, 783 WNV human cases were reported in Europe (Gray and Webb, 2014). In 2016, 2,038 cases of WNV disease in human were reported to USA Centers for Disease Control and Prevention (CDC), with 56% of the cases classified as neuroinvasive disease (CDC, https://www.cdc.gov/westnile/statsmaps/preliminarymapsdata/index.html, accessed July 2017). WNV is thus not likely to disappear on its own accord and requires further research to develop effective treatments.

To achieve this, it is important to understand WNV infection, which can either lead to mild symptoms, common to other febrile diseases or to a more severe clinical form of neuro-invasive disease that includes neck stiffness, stupor, disorientation, meningitis, paralysis, coma, and death (Rossi et al., 2010). Only 1% of the infection cases progress to this final neuro-invasive stage (Diamond, 2009; Kimura et al., 2010; Rossi et al., 2010; Lim et al., 2011b; Sejvar, 2014). Although uncommon, this neurological stage is life threatening. It is crucial to avoid WNV infection to evolve to such condition. For this, it is necessary to understand WNV infection progression. Typically, following a bite of a WNV infected mosquito, in the first stage the virus infects keratinocytes and Langerhans cells, which end up in regional lymph nodes, where the first round of the initial replication occurs (Johnston et al., 2000; Lim et al., 2011a). On a second stage, another round of replication occurs, when WNV titer becomes high enough for it to spread systemically to visceral organs, primarily infecting the kidney and the spleen (Johnston et al., 2000; Samuel and Diamond, 2005; Tesh et al., 2005; Lim et al., 2011a). The disease progresses to the neuro-invasive stage only if high viremia is achieved at this crucial stage (Samuel and Diamond, 2005; Tesh et al., 2005). Therefore, blocking the infection at the visceral stage is critical for preventing its evolution to the life-threatening neurological stage (Diamond, 2009; Kimura et al., 2010; Rossi et al., 2010; Lim et al., 2011b). For this to be possible, it is important to examine the similarities between WNV and closely related flaviviruses, especially in the first stages of infection.

Members of *Flavivirus* genus, *Flaviviridae* family, to which WNV belongs, are structurally similar, with homologous proteins sharing highly conserved regions. Flaviviruses such as WNV are icosahedral enveloped viruses composed of a lipid bilayer surrounding a nucleocapsid containing a positive sense single-stranded genomic RNA complexed with multiple copies of the capsid (C) protein (Mukhopadhyay et al., 2005; Bhuvanakantham and Ng, 2013). Viral assembly, one of the most important processes of the virus life cycle, is mediated by the C protein. The C proteins have roughly 100 amino acid residues. WNV, DENV, and ZIKV capsid proteins, for example, have 105, 100, and 104 residues, respectively, being highly similar, as previously reported by us (Martins et al., 2012). In solution, the C proteins of WNV and DENV form a homodimer rich in α-helices, with each monomer composed by four α-helices (named α1 to α4) connected by short loop regions (Jones et al., 2003; Dokland et al., 2004; Ma et al., 2004), which are similar in terms of sequence and structure (Martins et al., 2012). WNV and DENV C proteins have an asymmetric charge distribution, being very positively charged proteins, while also containing hydrophobic pockets. The first 20 residues of WNV C, as well of other *Flavivirus* C proteins, namely DENV, are intrinsically disordered and are expected to facilitate their functions (Ivanyi-Nagy and Darlix, 2010). Among them, C protein interaction with host lipid structures, essential for viral replication, is most relevant for future drug design (Martins et al., 2012; Faustino et al., 2014, 2015a).

Viruses from the *Flaviviridae* family manipulate the host lipid metabolism to induce the conditions needed for their own viral replication (Zhang et al., 2017). Lipid droplets (LDs) have been studied as important intracellular organelles for virus pathogenesis. LDs are formed in the endoplasmic reticulum (ER) and play a crucial role in cell homeostasis. Although LDs are used by the immune system against pathogens, some viruses have evolved strategies to use these organelles as platforms for viral assembly and replication. Moreover, viruses use LDs as energy reservoirs during replication of the viral genome, an energy-consuming process (Wang, 2016). For instance, DENV has been proposed to use LDs through the process of lipophagy, for viral replication (Heaton and Randall, 2010). Furthermore, LDs have been proposed as a platform for viral assembly (Roingeard and Melo, 2017). Importantly, LDs are targeted by several structural and non-structural viral proteins during the virus life cycle (Zhang et al., 2017). The association of these proteins with LDs has been studied in order to understand their role in the key intracellular processes that occur during virus assembly and replication. In some members of the *Flaviviridae* family, such as DENV and hepatitis C virus (HCV), the interaction of the homologous C proteins with host lipid systems, namely intracellular LDs and blood plasma very low-density lipoproteins (VLDL), was shown to be important for these viruses biological activity (Mukhopadhyay et al., 2005; Samsa et al., 2009; Carvalho et al., 2012; Martins et al., 2012; Faustino et al., 2014). Given that WNV and DENV C are closely related and highly similar (Mukhopadhyay et al., 2005; Martins et al., 2012) and that the interaction of DENV C with host LDs is essential for successful dengue viral replication (Samsa et al., 2009), such interaction may play a major role in WNV and related viruses. This led to studies on the interaction of host LDs with flaviviruses C proteins. Interestingly, ZIKV C protein was recently reported to co-localize with LDs as well (Shang et al., 2018). Moreover, DENV C-LDs interaction is strong and specific, requiring LDs surface proteins for the binding, as well as the characteristic high intracellular potassium concentrations (Carvalho et al., 2012). Importantly, the residues within DENV C hydrophobic α2–α2′ core involved in LDs interaction are conserved among flaviviruses, including WNV, both in terms of sequence and structural organization (Martins et al., 2012).

As no specific treatment is available for WNV infection, clarifying the biological activity of WNV C regarding its ability to interact with host lipid systems may pave the way for future treatments against this and related viruses. With this in mind, we first characterized relevant host lipid systems, namely LDs isolated from baby hamster kidney (BHK-21) cells and lipoproteins isolated from human blood plasma, in the absence of viral proteins. Following, we tested WNV C interaction with these lipid systems using zeta potential (ζ-potential) analysis, dynamic light scattering (DLS) and atomic force microscopy (AFM) based force spectroscopy. Our results show that WNV C binds to LDs and VLDL and that these interactions are strong and specific, involving K^+^ ions and surface proteins from these host lipid systems. The use of single-molecule methods to study and measure biologically relevant protein-ligand interactions is a promising avenue of research for nanomedicine, enabling the acquisition of detailed structural knowledge on the system under study. Here, we successfully applied such approaches at the level of an important human pathogen, by studying a key viral protein, the WNV C protein, in the context of its interaction with relevant host lipid systems. A structural understanding of the major factors modulating these key interactions of WNV C is extremely important, since it may lead to new drug development approaches against WNV and other flaviviruses, such as the closely related Dengue and Zika viruses.

## Materials and Methods

### Materials

Human plasma lipoproteins were obtained from Kalen Biomedical LLC (Montgomery Village, MD, USA). WNV C protein, serotype Kunjin, 105 residues (11.7 kDa), was chemically synthesized by VCPBIO (Shenzen, China) with >95% purity. The C-terminal was amidated and the N-terminal acetylated. The secondary structure of the protein was evaluated via circular dichroism spectroscopy, showing α-helical and random coil content.

Experiments were performed in two different buffers: TEE KCl buffer (20 mM Tris-HCl, 100 mM KCl, 1 mM EDTA and 1 mM EGTA, pH 7.4) and TEE NaCl buffer (20 mM Tris-HCl, 100 mM NaCl, 1 mM EDTA and 1 mM EGTA, pH 7.4).

### Cell Culture and Lipid Droplets Isolation

LDs were isolated and purified from baby hamster kidney (BHK-21) cells, by cell cavitation followed by sucrose-gradient ultracentrifugation, as previously described (Carvalho et al., 2012; Martins et al., 2012). Briefly, BHK-21 cell line was maintained in high-glucose Dulbecco's modified Eagle's medium (DMEM) with 0.01% sodium pyruvate and 4 mM L-glutamine, supplemented with 10% fetal bovine serum, 100 U/mL penicillin and 100 U/mL streptomycin in a T75 culture flask. Cells were grown at 37 °C, in a humidified 5% CO_2_ incubator. After 72 h, 24 h before LDs isolation and when approximately 80% confluence was reached, the medium was replaced for DMEM without fetal bovine serum and antibiotics, and cells were treated with 10 mM oleic acid. After 24 h of cells incubation with oleic acid, LDs were isolated by washing cells twice and disrupting them in TEE buffer with 100 mM KCl, centrifuging the cell lysate and collecting the supernatant, from which the LDs fractions were isolated via ultracentrifugation, following our previous work (Carvalho et al., 2012; Martins et al., 2012). LD fractions were kept at 4°C, checking before use if they present the previously reported ζ-potential value of, roughly, −20 mV (Carvalho et al., 2012). The same protocol was performed to isolate LDs in TEE NaCl buffer.

### DLS Measurements of Lipoproteins

Dynamic light scattering (DLS) measurements were carried out on a Malvern Zetasizer Nano ZS (Malvern, UK) equipped with a He-Ne laser (632.8 nm), with backscattering detection at 173°. DLS allows to measure particle hydrodynamic diameter and size distribution of molecules or supramolecular aggregates, based on the light scattering intensity fluctuation on a small volume, on a timescale of microseconds, due to the Brownian motion of the particles (Domingues et al., 2008; Stetefeld et al., 2016). The scattered light is collected and measured at a given angle by a sensitive detector. Size determinations can be performed through the measurement of the scattering light intensity fluctuations as a function of time. The size of particles in suspension can be determined in terms of *D*_*H*_, analyzing the normalized intensity autocorrelation functions. With the Stokes-Einstein equation, it is possible determine the hydrodynamic diameter (*D*_*H*_) from the diffusion coefficient (D) value (Berne and Pecora, 1990):

(1) $$\begin{array}{r} D_{H}=\frac{\kappa T}{3\pi \eta D} \end{array}$$

where η is the dispersant viscosity, κ the Boltzmann constant and *T* the absolute temperature. For these measurements, glass cuvettes with round aperture were used. VLDL or low-density lipoproteins (LDL) were independently diluted to a final concentration of 50 μg/mL in TEE KCl buffer. VLDL were also diluted in TEE NaCl buffer at the same final concentration. The *D*_*H*_ of both lipoproteins was measured without WNV C. Afterwards, successive volumes of WNV C solution were added, in order to assess concentrations from 0.25 to 5 μM, and the *D*_*H*_ was determine for each of them. Samples were allowed to equilibrate for 15 min at 25 °C before measurements. For each sample, 10 measurements were conducted, each measurement being the average of 10 runs of 10 s each, without waiting between them. This procedure was repeated at least three times for each condition, with independent lipoproteins samples. The normalized intensity autocorrelation functions were analyzed with the CONTIN method (Provencher, 1982). The *D*_*H*_ value of each measurement was obtained from the peak of the particle number distribution, *n*(*D*_*H*_), of each of the 10 measurements. Lipoproteins size data was analyzed intra-group (of 10 measurements) by average and standard deviation, discarding outliers. The average without outliers was close to the median in all the size data points. Values are presented as mean ± standard error (SE). Data sets were compared against the set of measurements without WNV C using the Mann-Whitney U test. Differences were considered statistically significant when *p* < 0.05.

### Zeta Potential Analysis of LDs Surface Charge

Zeta potential (ζ-potential) experiments were performed in the same equipment used for the DLS measurements (Malvern Zetasizer Nano ZS). ζ-potential measurements are based on the concept that charged particles in suspension attract to their surface ions with opposite charge, to which they can be strongly bound. These surface-bound ions form a layer, the Stern layer (Uskoković, 2012). Beyond the Stern layer, another layer is formed, where ions diffuse more freely. When the particle moves in the solution, the ions strongly attached to their surface move with it, whereas the ions in the diffuse boundary do not move with the particle. The potential that exists at this boundary is defined as the ζ-potential (Kirby and Hasselbrink, 2004; Domingues et al., 2008). The ζ-potential of the particles can be calculated using the Henry's relation (Domingues et al., 2008):

(2) $$\begin{array}{r} \zeta =\;\frac{3\eta u}{2\varepsilon f\left(ka\right)} \end{array}$$

where ζ is the ζ-potential, *u* the electrophoretic mobility, η the viscosity of the solvent, ε its dielectric constant and *f*(*ka*) is the Henry's function.

The physical constants used for the calculations were: *n*_0_ = 1.330, η = 0.8872 cP, *T* = 298.15 K, λ = 632.8 nm and θ = 13°. LDs samples were equilibrated for 15 min at 25 °C, at the Zetasizer, and then ζ-potential was determined from the average of 15 measurements (100 runs each), with 90 s of waiting time between measurements. Samples were analyzed by measuring independently the ζ-potential of LDs in a final volume of 842.5 μL, after incubation for 15 min at room temperature with different concentrations of WNV C. Following previous approaches (Carvalho et al., 2012; Martins et al., 2012), limited proteolysis of LDs with trypsin was performed by incubating the LDs samples with 10 μM trypsin in TEE buffer (with KCl or NaCl) for 15 min at room temperature. To stop the reaction, 1 mM phenylmethylsulfonyl fluoride (PMSF) was added to the mixture for 5 min at room temperature, after which the ζ-potential of trypsinized LDs samples was analyzed by measuring independently LDs in a final volume of 842.5 μL, after incubation for 15 min at room temperature with different concentrations of WNV C. The variation of zeta potential (Δζ) for each sample was determined by subtracting the value of the ζ-potential of LDs in the absence of WNV C from the ζ-potential of LDs in the presence each WNV C concentration. Experimental data was fitted using the equation:

(3) $$\begin{array}{r} \Delta \zeta =\;\frac{\Delta \zeta_{max}\left[WNV\;C\right]}{C_{1/2}+\left[WNV\;C\right]} \end{array}$$

where Δζ_max_ is the fitted maximum amplitude of variation of the ζ-potential induced by the interaction with WNV C, and *C*_1/2_ is the WNV C concentration at Δζ_max_/2. Light scattering spectroscopy techniques have been used in studies of different fields. Here, we used ζ-potential to study the interaction of the C protein with LDs. The same approach can be used to study charged particles, such as peptides, erythrocytes, and bacteria. For instance, ζ-potential measurements were employed to study the effect of erythrocytes aging on the interaction with fibrinogen (Carvalho et al., 2011). The same type of measurements were also used to evaluate the effect of antimicrobial peptides on lipid vesicles mimicking bacteria-like membranes (Irazazabal et al., 2019). Furthermore, ζ-potential has been used to characterize nanoparticles developed for biomedical application, namely to validate the electrostatic interaction between nanoparticles and their targets and to characterize the peptide anchoring profile to nanoparticles (Carvalho et al., 2018). DLS may complement these studies, providing quantitative information on particle size distribution. Besides determining the size distribution of nanoparticles, DLS measurements were used to confirm surface functionalization, characterize long term stability in different conditions and identify the aggregation profile of nanoparticles (Carvalho et al., 2018).

### LDs and Lipoproteins Preparation for Force Spectroscopy Measurements

Ten μL of LDs or human plasma lipoproteins (VLDL or LDL) suspensions were placed onto thin freshly cleaved muscovite mica and allowed to deposit for 30 min at room temperature. Non-adherent LDs or lipoproteins were removed by 5 sequential washing steps with TEE buffer (with Na^+^ or K^+^, depending on the experiment). Samples were loaded into the AFM apparatus and allowed to equilibrate in the respective TEE buffer for 10 min before force spectroscopy measurements.

### Functionalization of AFM Tips With WNV C

A protocol well established in our lab was used to functionalize AFM tips with WNV C for force spectroscopy measurements (Carvalho and Santos, 2012; Carvalho et al., 2012; Faustino et al., 2015b; Guedes et al., 2016). OMCL TR-400-type silicon nitride tips (Olympus, Japan) were cleaned with an intense UV light source and silanized in a vacuum chamber with 3-aminopropyl-triethoxysilane (APTES, 30 μL) and *N*,*N*-di-isopropylethylamine (10 μL), for 1 h, under an argon atmosphere, to be coated with a self-assembled monolayer of amines. Following this, probes were rinsed with fresh chloroform and dried with nitrogen gas. The silanization process results in a uniformly distributed self-assembled monolayer of amino-terminated APTES molecules on the AFM tips, which were then placed into a 2.5% (v/v) glutaraldehyde solution for 20 min and washed 3 times with TEE buffer. Finally, the tips were placed into a 187 μM WNV C solution during 30 min to covalently bind the protein. Protein-functionalized tips were immediately mounted onto the AFM instrument and used for the force spectroscopy measurements.

### AFM-Based Force Spectroscopy Measurements

AFM measurements were performed with a NanoWizard II atomic force microscope (JPK Instruments, Berlin, Germany), mounted on top of an Axiovert 200 inverted microscope (Zeiss, Jena, Germany), using triangular cantilevers with a pyramidal tip with radius of 15 nm and a resonance frequency of 11 kHz in air (OMCL-TR400, Olympus Europe, Germany). The AFM head is equipped with a 15-μm z-range linearized piezoscanner and an infrared laser. The spring constant of the tips were calibrated by the thermal fluctuation method, yielding values of 22 ± 5 mN/m. For each contact of the cantilever with LDs or plasma lipoproteins, the AFM tip-sample distance was adjusted in order to maintain an applied force of 200 pN before retraction. Molecular recognition was searched by pressing the tip intermittently onto different points of LDs or lipoproteins adsorbed to the mica surface. Data were collected for each force-distance cycle at 2 μm/s, leading to a loading rate of 4 nN/s. Experiments were performed at room temperature, by maintaining the laboratory between 23 and 25 °C. Small variations in temperature at this range did not affect the force spectroscopy measurements. In the experiments with LDs, measurements were conducted using TEE buffer with KCl (10 or 100 mM) or NaCl (100 mM). In the experiments with lipoproteins, measurements were conducted using TEE buffer with 100 mM KCl, both for VLDL and LDL samples, and using TEE buffer with 100 mM NaCl for VLDL. Each experiment was performed at least three times, each time on different samples and with different functionalized tips. For any given experiment, approximately 5,000 force-distance curves were collected and analyzed (Carvalho et al., 2010, 2012, 2013; Martins et al., 2012; Faustino et al., 2014). Force curves were analyzed using the JPK image processing software v. 4.2.61 (JPK Instruments, Berlin, Germany). Histograms of the (un)binding forces of each studied protein-LD or protein-lipoprotein interaction were constructed choosing the ideal bin size to achieve the best-fitted Gaussian model peak forces. The selected binning size was 6 pN. Force rupture values ranging between 0 and 10 pN were considered to represent noise or experimental artifacts, while values up to 25 pN were assigned to unspecific interactions (Carvalho et al., 2012; Faustino et al., 2014). From each histogram, the most likely single WNV C-host lipid system rupture force can be determined by fitting the distributions of the rupture forces with the Gaussian model. Measurements with tips at different steps of the functionalization process (including non-functionalized tips) were conducted on mica, and on LDs or lipoproteins samples, which serve as controls for the AFM tip functionalization process.

AFM-based force spectroscopy was used in this study to measure the interaction forces between molecules. Taking advantage of its piconewton sensitivity, we measured the force necessary to break the bonds between WNV C and LDs or lipoproteins. The same approach was used to study other molecular interactions (Guedes et al., 2016). AFM is commonly used to construct topographical images of the surface of a sample by scanning or tapping the sample surface with a tip mounted under a flexible cantilever. A laser beam is reflected on the back of the cantilever and any small deflections are amplified by an optical lever mechanism, using as detector a position-sensitive photodiode (Carvalho et al., 2013). These deflections are processed by the electronic system and the sample surface topography is determined (Carvalho et al., 2013). AFM provides detailed information of a sample surface properties. For instance, AFM has been extensively used to study the effect of antimicrobial peptides/proteins on human pathogens (Domingues et al., 2014; Migliolo et al., 2016; Gonçalves et al., 2017).

## Results

To unravel the details of WNV C interaction with relevant host lipid systems, a combination of biophysics techniques was employed. ζ-potential studies and AFM-based force spectroscopy (un)binding analysis were performed to characterize WNV C interaction with LDs, quantifying the role of charges in the interactions, the binding affinity, as well as the binding forces of the interactions at the single-molecule level. DLS and AFM-based force spectroscopy measurements were combined to characterize the WNV C interaction with plasma lipoproteins, namely VLDL and LDL. By combining these different techniques, it was possible to determine the single-molecule level interaction forces between WNV C and VLDL and to observe the increment in VLDL hydrodynamic diameter upon the interaction with WNV C.

### ζ-Potential Measurements Demonstrate a WNV C-LDs Binding Influenced by K^+^ and LDs Surface Proteins

**Figure 1:**
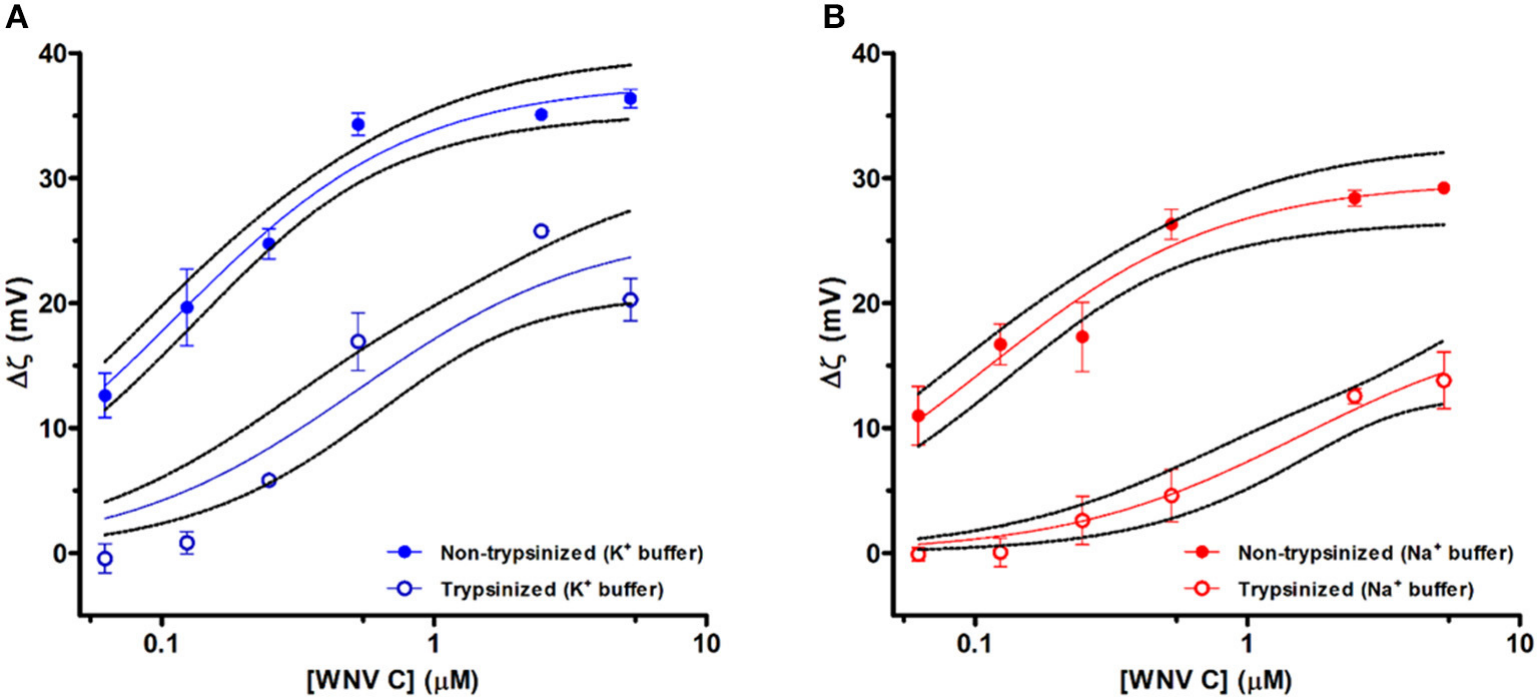
LDs ζ-potential analysis at different WNV C concentrations. Variation of ζ-potential (Δζ) values of LDs samples isolated with TEE KCl buffer (K^+^ buffer) **(A)** and TEE NaCl buffer (Na^+^ buffer) **(B)** in the absence and in the presence of distinct WNV C concentrations. ζ-potential values determined for LDs without trypsin preincubation (filled symbols) and LDs pre-incubated with trypsin (empty symbols). WNV C concentration axes are presented in logarithmic scale. Results are presented as mean ± standard error (SE). Experiments were performed in triplicate. Solid lines were obtained by fitting the experimental data using Equation 3. WNV C binds to LDs inducing a progressive increase in the surface charge of the LD-WNV C complex.

ζ-potential measurements were performed to determine if WNV C is able to bind to LDs. Upon the addition of WNV C to LDs in TEE buffer with 100 mM KCl, there was a concentration dependent increase in the ζ-potential (Figure 1A; data are presented as the variation of ζ-potential of LDs in the absence of WNV C and LDs in the presence of WNV C, Δζ). In the absence of WNV C, LDs in TEE buffer with 100 mM KCl present a negative ζ-potential value (−20.6 ± 0.7 mV). The titration of LDs suspension with WNV C induced a progressive increase in the scattering particle charge, stabilizing at positive values (+15.8 ± 0.7 mV for a WNV C concentration of 5 μM). With an identical addition of WNV C to trypsinized LDs, there was a lower increase in Δζ than for the non-trypsinized LDs (Figure 1A). Trypsinized LDs have an initial value of −18.8 ± 1.2 mV that, upon titration, increases and stabilizes at +1.5 ± 1.7 mV. The increase in Δζ of non-trypsinized LDs is much higher than the observed for trypsinized LDs at similar concentrations. The replacement of potassium ions on the TEE buffer by the same concentration of sodium ions yields similar results. Values of ζ-potential for LDs in TEE NaCl buffer also increase for higher WNV C concentrations (Figure 1B). In this sodium buffer, LDs present a ζ-potential of −16.5 ± 0.5 mV in the absence of WNV C. At the highest WNV C concentration tested (5 μM), the ζ-potential value reaches +12.7 ± 0.3 mV. The addition of WNV C to trypsinized LDs in TEE NaCl buffer lead to a lower increase of LDs ζ-potential than the non-trypsinized LDs (Figure 1B), from −16.6 ± 0.6 mV (without WNV C) up to −2.8 ± 2.3 mV (at the highest concentration of WNV C). Overall, Δζ is consistently smaller in sodium buffer than for the potassium buffer to the same WNV C concentration.

The experimental Δζ-potential WNV C-LDs binding curves were fitted to an empirical binding curve model (Carvalho et al., 2012) (Figure 1). The values of the maximum amplitude of variation of ζ-potential induced by the interaction with WNV C (Δζ_max_) and the WNV C concentration at Δζ_max_/2 (the half-maximal effect, *C*_1/2_) are presented on Table 1. Non-trypsinized LDs in TEE KCl buffer display a *C*_1/2_ of 112 ± 15 nM while for trypsinized LDs a 4.6-fold higher *C*_1/2_ value (519 ± 168 nM) is observed. LDs surface proteins are therefore important for the interaction with the viral protein. K^+^ ions also seem to play a role in WNV C-LDs interaction. When TEE KCl buffer is replaced with TEE NaCl buffer, a *C*_1/2_ of 111 ± 21 nM for non-trypsinized LDs is observed. However, if the same experiment is performed with trypsinized LDs a *C*_1/2_ of 1560 ± 697 nM (14-fold higher) is observed. Moreover, the difference between the fitted Δζ_max_ values for the several conditions tested also worth note. The Δζ_max_ value for non-trypsinized LDs in TEE KCl buffer (+37.6 ± 1.2 mV) is higher than the Δζ_max_ for trypsinized LDs in the same buffer (+26.0 ± 2.5 mV), and it is also higher than the Δζ_max_ for non-trypsinized LDs in TEE NaCl buffer (+29.8 ± 1.5 mV). Just focusing on TEE NaCl buffer, the non-trypsinized LDs Δζ_max_ value is also higher than the Δζ_max_ for the trypsinized LDs (+18.8 ± 3.1 mV). Therefore potassium ions seems to play a role in WNV C interactions with LDs, in line with previous findings for DENV C protein (Carvalho et al., 2012).

**Table 1:** ζ-potential analysis of LDs titration with WNV C.

**Condition**		***C*_**1/2**_ (nM)**	**Δζ**_**max**_ (mV)
K^+^ buffer	Non-trypsinized	112 ± 15	37.6 ± 1.2
	Trypsinized	519 ± 168	26.0 ± 2.5
Na^+^ buffer	Non-trypsinized	111 ± 21	29.8 ± 1.5
	Trypsinized	1560 ± 697	18.8 ± 3.1

*The values shown (presented as mean ± SE) are the maximum amplitude of variation of the LDs ζ-potential (Δζ_max_) induced by the interaction with WNV C, and the WNV C concentration at Δζ_max_/2 (C_1/2_). Values of C_1/2_ and Δζ_max_ were obtained through the fitting of ζ-potential experimental data using equation 3. K^+^ and Na^+^ buffers are TEE buffer with 100 mM KCl or 100 mM NaCl, respectively*.

### AFM WNV C-LDs Interaction Studies Corroborate ζ-Potential Data

**Figure 2:**
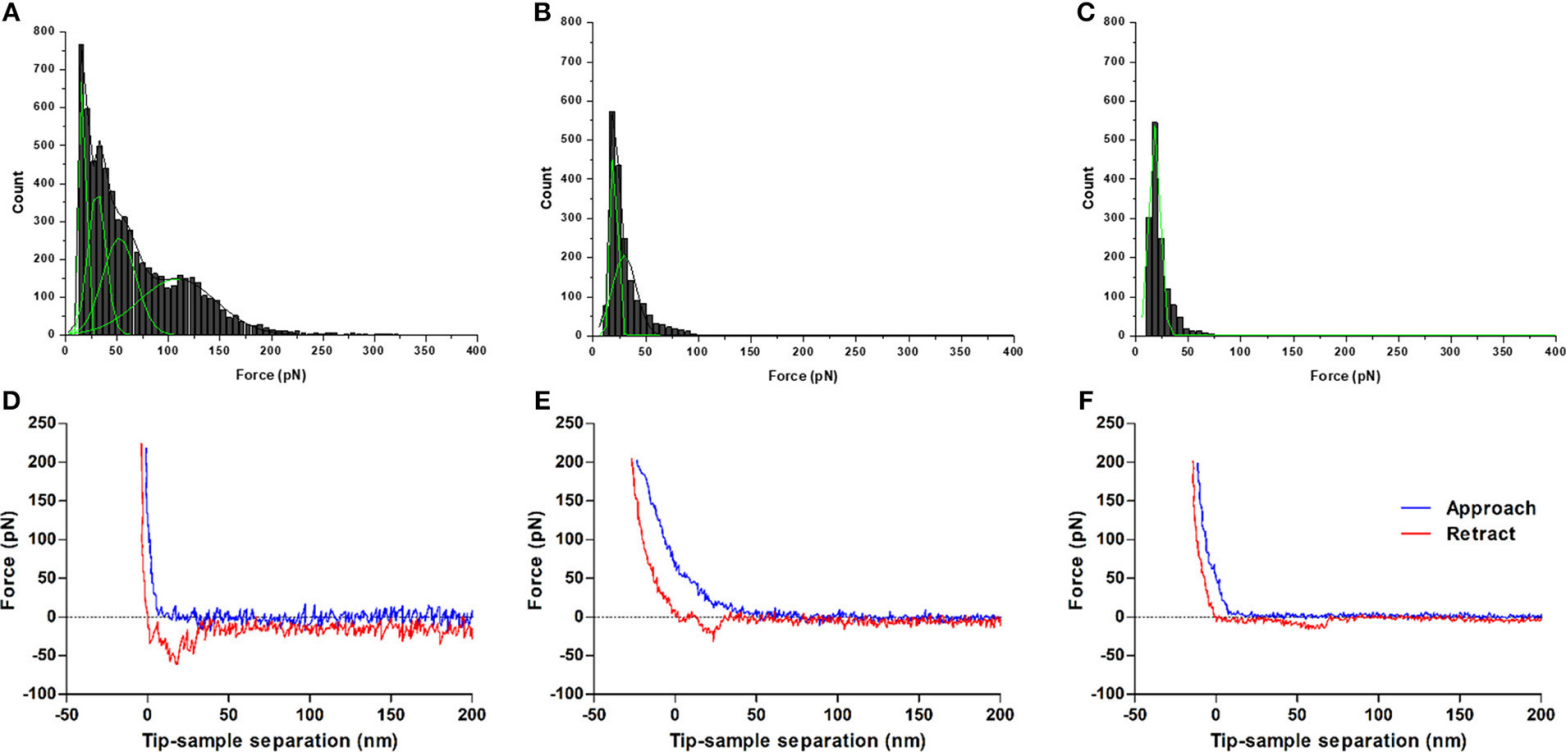
Force rupture histograms of WNV C-LDs binding, obtained by AFM-based force spectroscopy. Experiments were performed in TEE buffer with 100 mM KCl **(A)**, 10 mM KCl **(B)** or 100 mM NaCl **(C)**. The stronger and specific interaction forces between WNV C and LD are only found at K^+^ concentrations similar to the intracellular environment. Only weak interactions, characteristic of unspecific binding, are observed for low potassium ion concentrations or in its absence. Gaussian fitting lines of each individual peak (green) and cumulative fit lines of the histogram (black) are presented. Examples of approach-retraction curves acquired during the force spectroscopy measurements of WNV C-LDs adhesion in TEE buffer with 100 mM KCl **(D)**, 10 mM KCl **(E)**, or 100 mM NaCl **(F)**.

Single-molecule AFM-based force spectroscopy was employed to measure the specific interactions between WNV C and LDs. (Un)Binding forces were measured based on the deflection of AFM tips functionalized with WNV C and allowed to interact with LDs (Figures 2D–F). Figures 2A–C shows the force histograms obtained for the binding and subsequent unbinding between the WNV C-functionalized AFM tip and LDs in the presence of different potassium chloride concentrations. The distribution of the length of the rupture adhesion events between WNV C and LDs was analyzed by fitting the obtained histogram with the Gaussian model described in material and methods. A force rupture value of 30.4 ± 0.3 pN was determined at 100 mM KCl (Figure 2A). This value corresponds to the single-molecule interaction force necessary to break the bond between one WNV C protein dimer and one LD. In line with previous observations, a peak with weaker interaction forces is found (≈ 17.7 pN), which is attributed to unspecific interactions (Carvalho et al., 2012). The two other peaks of stronger interaction forces (52.0 ± 2.5 pN and 108.5 ± 2.8 pN) likely correspond to the rupture of multiple bonds due to the interaction of more than one protein dimer attached to the tip with a LD. As such, a clear, strong and specific binding of WNV C to LDs occurs in the presence of potassium ions.

To further evaluate the role of K^+^, its concentration in the buffer was decreased from 100 to 10 mM (Figure 2B). In this condition, a significant decrease in the (un)binding frequency is seen, from 62.7% in TEE buffer with 100 mM KCl to 19.4% in TEE buffer with 10 mM KCl (Table 2). At the low potassium concentration, the force histogram also changes significantly: only two force peaks are observed, at 20.1 ± 0.2 pN and 29.6 ± 0.9 pN. These peaks are similar to the first two observed in TEE buffer with 100 mM KCl (≈ 17.7 pN and 30.4 ± 0.3 pN). The weaker forces peak possibly corresponds to unspecific interactions and the stronger to the force necessary to break the WNV C-LDs binding. Higher interaction forces, corresponding to the rupture of multiple bonds, are not observed at low potassium concentrations. When force spectroscopy measurements are performed replacing the potassium by 100 mM sodium (Figure 2C), a dramatic change occurs, with a single rupture force peak of 17.9 ± 0.1 pN being observed, typical of unspecific interactions. In agreement with this, a low (un)binding frequency of 19.3% is seen for that condition (Table 2). Therefore, the interaction between WNV C and LDs is K^+^-dependent, rather than a general dependence of ionic strength.

**Table 2:** Rupture forces and percentage of (un)binding events obtained by AFM-based force spectroscopy for the interaction between WNV C and LDs.

**Experimental condition**		**% (Un)binding events**	**Rupture Force (pN)**			
			**1st peak**	**2nd peak**	**3rd peak**	**4th peak**
[KCl]	100 mM	62.7	≈ 17.7	30.4 ± 0.3	52.0 ± 2.5	108.5 ± 2.8
	10 mM	19.4	20.1 ± 0.2	29.6 ± 0.9		
[NaCl]	100 mM	19.3	17.9 ± 0.1			

*Values are presented as mean ± SE*.

### DLS Shows That WNV C Interacts With VLDL but not With LDL in a K^+^-Dependent Manner

**Figure 3:**
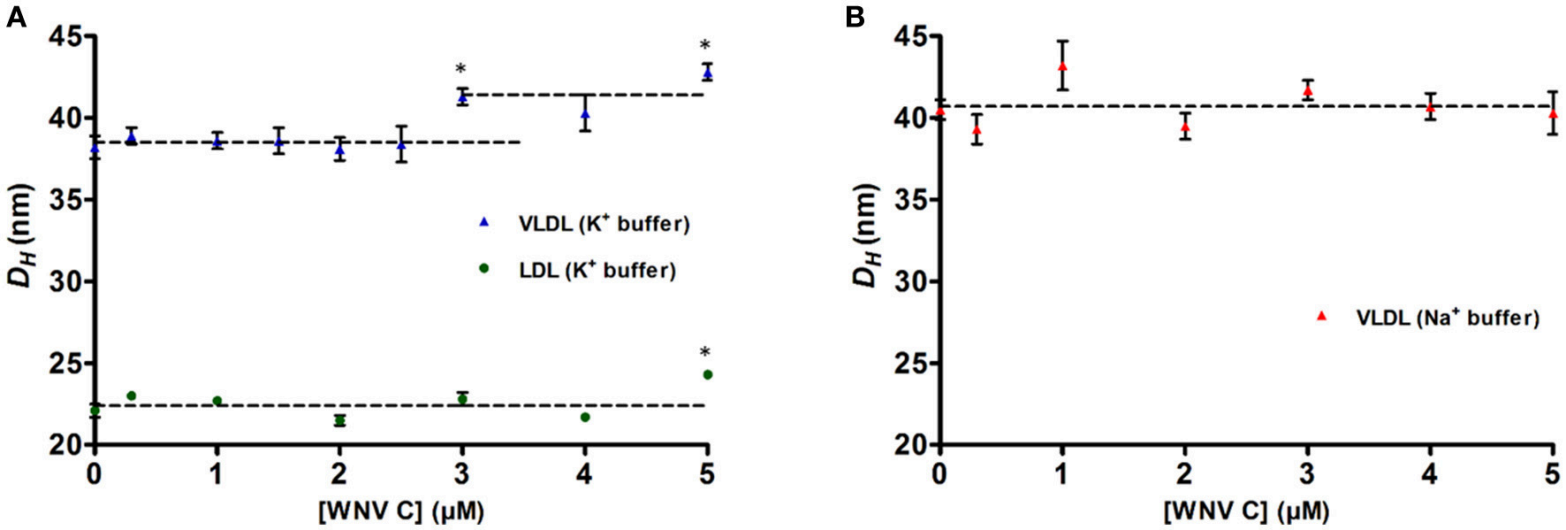
DLS analysis of lipoproteins hydrodynamic diameters upon titration with WNV C. In TEE buffer with 100 mM KCl, VLDL (blue triangles) average hydrodynamic diameter (*D*_*H*_) increases 4.5 ± 0.6 nm in the presence of WNV C, but no significant variation is observed for LDL (green circles) **(A)**. In TEE buffer with 100 mM NaCl, the *D*_*H*_ of VLDL (red triangles) does not change significantly upon increasing WNV C concentration **(B)**. K^+^ is therefore crucial for WNV C-VLDL interaction. *D*_*H*_ values (presented as mean ± SE) are the average of three independent measurements for each point (*p < 0.005). Dashed lines correspond to the average of each set of results.

Having established that WNV C binds LDs in a potassium specific manner that requires LDs surface proteins, the WNV C interaction with lipoproteins was then tested. DLS was employed to measure the scattered light intensity fluctuations of lipoproteins size that occur due to their Brownian motion, and calculate their hydrodynamic diameter (*D*_*H*_) using the Stokes-Einstein equation (Faustino et al., 2014). The average lipoproteins size in TEE buffer with 100 mM KCl was determined while they were titrated with WNV C. An increase in the VLDL size upon titration with WNV C is clearly seen (Figure 3A). DLS data shows that in the absence of WNV C, VLDL and LDL present average hydrodynamic diameters (*D*_*H*_) of, respectively, 38.2 ± 0.7 nm and 22.1 ± 0.4 nm (values are mean ± SE), in agreement with literature data (Cushley and Okon, 2002; Faustino et al., 2014). Upon titration with WNV C, a statically significant increase in the average size of VLDL occurs, up to 42.8 ± 0.5 nm (*p* < 0.005) at the maximum WNV C concentration tested. The VLDL *D*_*H*_ in the presence of WNV C has a 4.5 ± 0.6 nm increase relative to the value in the absence of the protein. By the observation of the intensity distribution, *I*(*D*_*H*_), of these measurements, it is possible to say that these values do not correspond to VLDL aggregation (that may occur in a small fraction of the total particles), but to an increase in the average size of VLDL due to the interaction with WNV C and formation of a WNV C-VLDL complex. In the case of LDL, upon titration with WNV C there was no clear change in *D*_*H*_ (Figure 3A).

Potassium ions, found at higher concentrations inside cells but at low concentration outside, were previously shown to be essential for DENV C-VLDL interaction (Faustino et al., 2014). With this in mind, to test the importance of K^+^ for the WNV C-VLDL binding, the size of VLDL and of their complex with WNV C was measured replacing TEE KCl buffer by TEE NaCl buffer (Figure 3B). VLDL in TEE NaCl buffer have a *D*_*H*_ initial value (40.5 ± 0.6 nm) higher than the observed in TEE KCl buffer. Upon titration with WNV C, there is no significant difference in *D*_*H*_, suggesting that in the absence of potassium ions WNV C cannot interact with VLDL. These results indicate that K^+^ is crucial for WNV C-VLDL binding.

**Figure 4:**
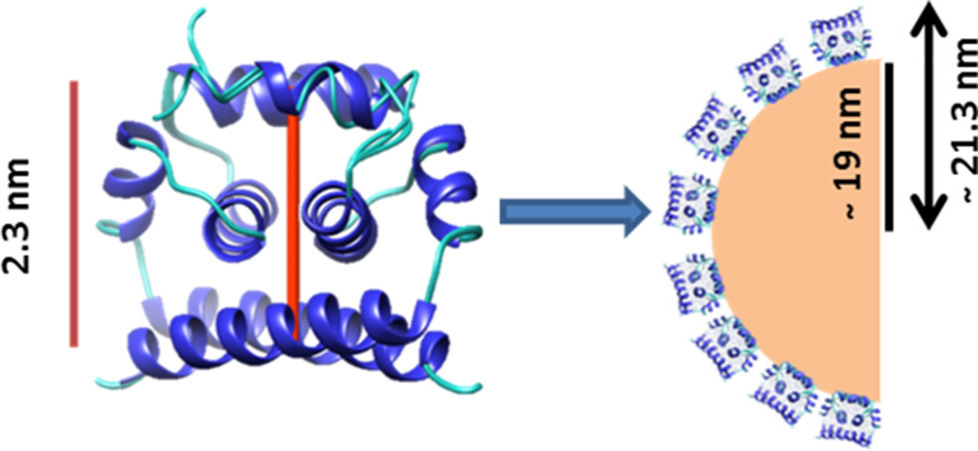
Proposed model of WNV C interaction with VLDL. WNV C dimensions fit well with the observed increase of ~2.3 nm on the VLDL hydrodynamic radius. Without WNV C, VLDL present a hydrodynamic radius of ~19 nm, while with 5 μM WNV C it increases to ~21.3 nm. Therefore, a monolayer of WNV C molecules could be formed on the VLDL surface.

Having determined the average increase in size of VLDL in the presence of high WNV C protein concentrations, this information was used to generate a model of WNV C binding to VLDL. Based on DLS data and on the dimensions of the protein molecule obtained from the structure 1SFK deposited at the Protein Data Bank (Dokland et al., 2004), we could elaborate a model for WNV C-VLDL interaction, whereby WNV C dimers bind to the VLDL surface, forming a single viral protein layer (Figure 4). VLDL hydrodynamic radius is ~ 19 nm. When WNV C interacts with its surface, the radius increases to ~ 21.3 nm. This 2.3 nm increment in radius correlates with the dimension of the WNV C dimer and is in total agreement with what would be expected for the formation a layer of WNV C on the surface of VLDL (Figure 4).

### AFM Data Supports WNV C-VLDL Specific and K^+^-Dependent Binding

**Figure 5:**
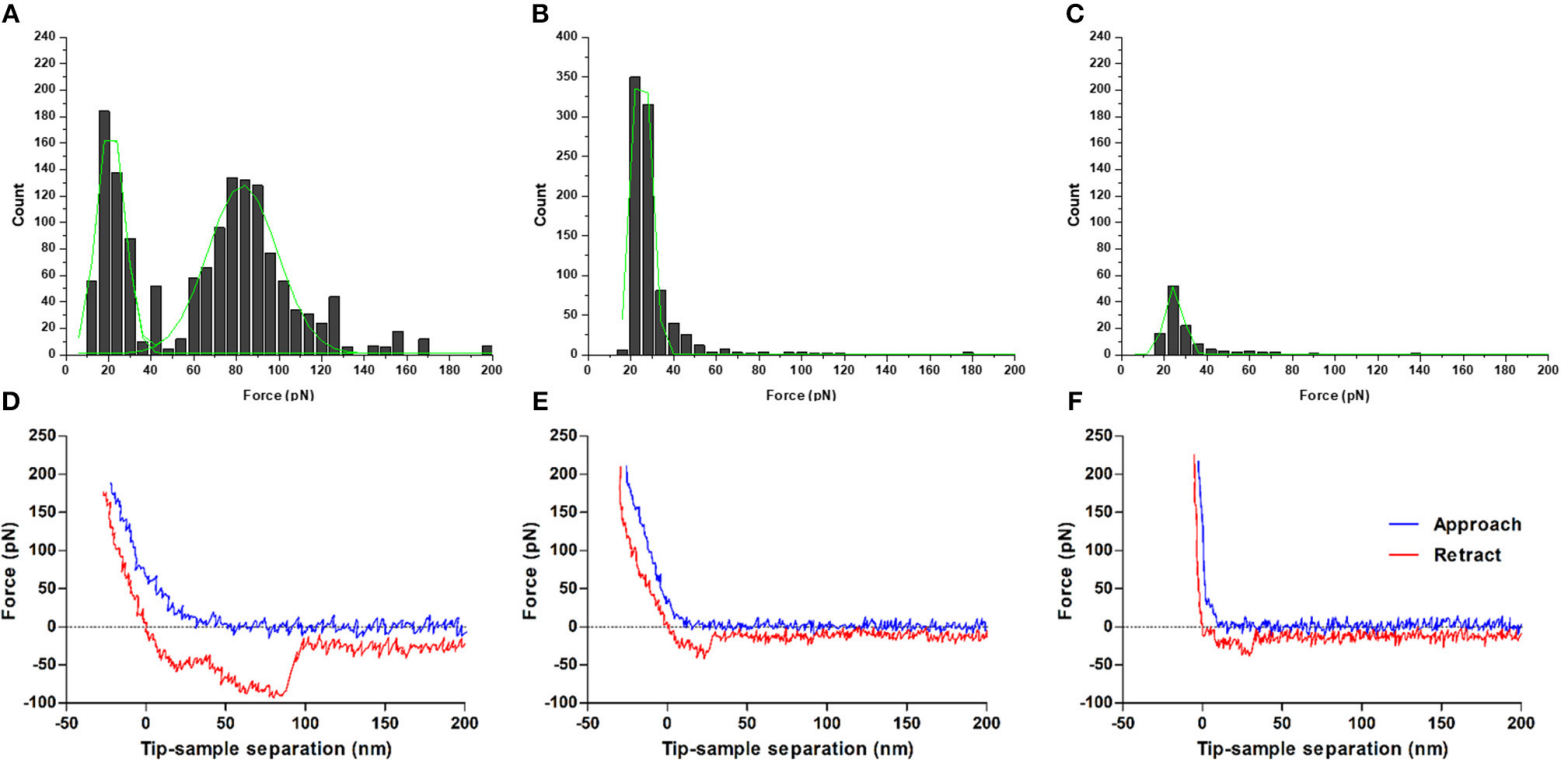
Force rupture histograms of WNV C interaction with plasma lipoproteins, obtained by AFM-based force spectroscopy. Experiments were performed in TEE buffer with 100 mM KCl for VLDL **(A)** and LDL **(B)**, as well as in TEE buffer with 100 mM NaCl for VLDL **(C)**. The single peak of weak rupture forces observed in **(B)** and **(C)** correspond to unspecific interactions. Strong and specific interactions only occur for the interaction of WNV C with VLDL in the presence of potassium ions. Gaussian fitting lines of each individual peak are shown in green. Examples of approach-retraction curves acquired during the force spectroscopy experiments of WNV C interaction with plasma lipoproteins in TEE buffer with 100 mM KCl for VLDL **(D)** and LDL **(E)**, as well as in TEE buffer with 100 mM NaCl for VLDL **(F)**.

To further complement the DLS data, single-molecule AFM-based force spectroscopy was employed to assess WNV C binding to human plasma lipoproteins (VLDL and LDL) (Figures 5D–F). As reported before (Faustino et al., 2014), the peak of forces around 20 pN is attributed to unspecific interactions, since it also appeared in the controls performed with non-functionalized tips and lipoproteins. Through analysis of the histograms, only forces above 40 pN were considered strong enough to report specific binding. The distribution of the force of the rupture adhesion events between WNV C and VLDL or LDL were analyzed by fitting the obtained histograms with the Gaussian model. Figures 5A–C presents the histograms of WNV C interaction with VLDL and with LDL, both in the presence of TEE buffer with 100 mM KCl. Comparing the histograms for VLDL and LDL, different peaks can be distinguished, corresponding to different rupture forces. In the VLDL histogram (Figure 5A), beside the unspecific interactions (21.0 ± 0.3 pN), strong and specific interactions are observed (82.9 ± 0.7 pN). The LDL histogram (Figure 5B) shows only one peak, with weak forces (24.5 ± 0.1 pN), characteristic of unspecific interactions. Moreover, as presented in Table 3, the (un)binding frequency registered for VLDL (31.1%) is much higher than for LDL (3.1%). As such, it is clear that WNV C binds specifically to VLDL and not to LDL, corroborating the DLS data.

**Table 3:** Rupture forces and percentage of (un)binding events determined with AFM-based force spectroscopy for the interaction between WNV C and VLDL or LDL.

**Experimental condition**	**% (Un)binding events**	**Rupture forces (pN)**	
		**1st peak**	**2nd peak**
**KCl 100 mM**			
VLDL	31.1	21.0 ± 0.3	82.9 ± 0.7
LDL	3.1	24.5 ± 0.1	
**NaCl 100 mM**			
VLDL	15.1	25.0 ± 0.1	

*Values are presented as mean ± SE*.

To determine if WNV C-VLDL binding requires potassium ions, as already demonstrated for WNV C-LDs binding, the interactions were also measured in the presence of TEE buffer with 100 mM NaCl (Figure 5C). Interestingly, the strong and specific interactions that occur in TEE buffer with 100 mM KCl disappear when the ionic strength is maintained but potassium ions are replaced for sodium ions. In the histogram, only a peak of weak forces is observed (25.0 ± 0.1 pN), corresponding to unspecific interactions. In agreement with these results, the (un)binding frequency also decreases significantly to 15.1% (Table 3). Therefore, AFM-based force spectroscopy also indicates that potassium ions are required for the WNV C-VLDL binding to occur.

## Discussion

The results obtained with ζ-potential measurements show that LDs in 100 mM KCl present, as expected, a negative surface charge, with an average ζ-potential of −20.6 ± 0.7 mV, which increases in the presence of WNV C (Figure 1 and Table 1). Single-molecule AFM-based force measurements give further support to these findings. WNV C-LD binding is strong and characteristic of specific binding, with a (un)binding force of 30.4 ± 0.3 pN (Figure 2 and Table 2). Both the AFM and the ζ-potential data are in excellent agreement with similar observations regarding DENV C-LDs binding (Carvalho et al., 2012). Moreover, the results show that WNV C-LDs interaction requires K^+^ concentrations similar to the intracellular medium and proteins present on LDs surface. LDs contain several proteins on their surface, majorly the proteins of PAT family, namely, perilipin 1 (formerly known just as perilipin), perilipin 2 (also known as ADRP), perilipin 3 (PLIN3; also known as TIP47), as well as other proteins in minor quantities (Olofsson et al., 2009). Considering the previous studies on DENV C-LDs binding (Carvalho et al., 2012), PLIN3 is the most likely target of WNV C in the LDs surface. Surprisingly, upon incubation of LDs with increasing concentrations of DENV C (Carvalho et al., 2012) or WNV C the values of *C*_1/2_ and of Δζ_max_ are comparable (LDs binding to WNV C: *C*_1/2_ = 112 ± 15 nM, Δζ_max_ = 37.6 ± 1.2 mV and LDs binding to DENV C: *C*_1/2_ = 85.7 ± 17.6 nM, Δζ_max_ = 34.4 ± 1.3 mV), suggesting that these two capsid proteins, besides being similar, may also bind to the same molecular target. Reinforcing these observations, the specific force peak around 30 pN (WNV C-LDs rupture force = 30.4 pN and DENV C-LDs rupture force = 34 pN) and the (un)binding frequency derived from force spectroscopy measurements are also similar between DENV C (Carvalho et al., 2012) and WNV C interacting with LDs (WNV C-LDs (un)binding frequency = 62.7 % and DENV C-LDs (un)binding frequency = 58.4 %). The requirement of potassium ions for WNV C to bind LDs, observed both *via* ζ-potential (Figure 1 and Table 1) and AFM-based force spectroscopy measurements (Figure 2 and Table 2), was found for DENV C-LDs interaction too (Carvalho et al., 2012), which further corroborates the similarities between the two viruses at this level. It was reported that related viruses require K^+^ for their infection processes (Griffin et al., 2003; Mankouri et al., 2009; McLauchlan, 2009). In fact, when viruses invade the host cells, the infectious process modulates their biochemistry and physiology. These mechanisms are frequently essential for the viral life cycle (Wang et al., 2011). We may hypothesize that beside WNV C binding to protein(s) on the surface of intracellular LDs, other *Flavivirus* C proteins may interact specifically with proteins on the LDs surface through a similar mechanism, essential for the viral replication process.

DLS and AFM-based force spectroscopy measurements clearly show that WNV C is able to interact specifically with VLDL in a potassium dependent manner, but not with LDL, as previously reported for DENV C (Faustino et al., 2014). DENV and WNV C proteins-VLDL interactions lead to increments of VLDL *D*_*H*_ of ~ 6 and 4.5 nm, respectively. Furthermore, AFM-based force spectroscopy data show two rupture force peaks for C protein-VLDL binding and one for LDL in the presence of 100 mM of potassium ions. The first peak observed corresponds to weak unspecific interactions and the second peak corresponds to strong and specific interactions between the C protein and VLDL. The same was previously observed for DENV C (Faustino et al., 2014). However, DENV C-VLDL specific rupture force (50.5 ± 0.5 pN) is lower than the observed for WNV C-VLDL rupture force peak (82.9 ± 0.7 pN), suggesting that WNV C interaction with VLDL may be stronger than for DENV C. Nevertheless, the frequency (probability) of DENV and WNV C (un)binding events obtained in the AFM tip approach/ retraction cycles were very similar (30.5% for DENV C and 31.1% for WNV C). WNV C only binds to VLDL in the presence of K^+^. When 100 mM potassium ions are replaced by 100 mM sodium ions, the rupture force between the C proteins (DENV C or WNV C) and VLDL are weaker, corresponding to unspecific interactions. Accordingly, in the presence of Na^+^, the percentage of (un)binding events changes from 30.5 to 10.7% for DENV C and from 31.1 to 15.1% for WNV C.

On the endogenous plasma lipoproteins cycle, in order to be converted in LDL, VLDL loose triacylglycerols, being enriched in cholesterol esters, become smaller in size and, additionally, they lose some of the VLDL intrinsic proteins. At the final stage, apolipoprotein B100 is the most important apolipoprotein in the mature LDL, while apolipoprotein E (APOE) is almost absent from their surface (Cushley and Okon, 2002). Interestingly, it was reported that DENV C interaction with LDs and VLDL occurs through the binding to PLIN3 (Carvalho et al., 2012) and APOE (Faustino et al., 2014), respectively. In this way, it may be expected that, as for DENV C, WNV C-VLDL interaction involves APOE. The formation of lipoviroparticles (LVPs) has been reported in viruses from the *Flaviviridae* family, such as HCV (Bartenschlager et al., 2011) and hepatitis G virus (Agnello et al., 1999), which enter host cells through the low-density lipoprotein receptor pathway (Agnello et al., 1999). In agreement with this hypothesis, it is known that plasma lipoproteins levels in circulation are greatly affected in *Flavivirus* infections (van Gorp et al., 2002; Suvarna and Rane, 2009; Bartenschlager et al., 2011), in particular in the most severe cases of dengue infection, for which formation of LVPs had been hypothesized (Faustino et al., 2014). WNV C association with VLDL suggests that WNV may also form LVPs. In fact, as observed for DENV-infected cells, WNV-infected cells present higher levels of unsaturated phosphatidylcholine species (Martín-Acebes et al., 2014), which may be involved in the development of more fluid membranes (Perera et al., 2012). However, the lipid composition of the WNV envelope differs from the cellular membranes. The envelope presents less phosphatidylcholine and higher levels of sphingomyelin (Martín-Acebes et al., 2014). WNV envelope differs from HCV envelope, for which it was reported the formation of LVPs (Bartenschlager et al., 2011). Further studies are necessary to understand how WNV C binding to VLDL contributes to viral pathogenesis.

In conclusion, WNV C interaction with LDs and VLDL is dependent on the concentration of potassium ions. In the presence of the same concentration of sodium ions, the specific C protein-host lipid systems interactions do not occur. Besides the dependence on potassium concentration, WNV C seems to interact with surface proteins of LDs and VLDL (probably PLIN3 and APOE, respectively). Since, *Flavivirus* C proteins are very similar in terms of sequence and structure, and it was reported before that DENV C interacts similarly to WNV C with host lipid systems, this could be a process common to all flaviviruses, namely the closely related Zika virus, which displays a highly homologous C protein. The biophysical and nanotechnology techniques described in this study, namely ζ-potential, DLS and AFM-based force spectroscopy may be employed to characterize important biological lipid systems in viral infection, as well as its interaction with the C protein of different viruses or with other proteins. Based on the characterization of WNV C-host lipid systems interactions, it is possible to develop new inhibitor peptides to block a key step of viral replication. Moreover, the existent inhibitors and those that may be developed can be tested with the approaches used in the present study to determine their ability to inhibit key interactions of the viral life cycle.

## Data Availability Statement

All datasets generated for this study are included in the manuscript.

## Author Contributions

FC, IM, and NS designed the experiments. AM, FC, AF, and IM performed the experiments. All authors participated in data analysis and writing of the manuscript.

### Conflict of Interest Statement

The authors declare that the research was conducted in the absence of any commercial or financial relationships that could be construed as a potential conflict of interest.
